# Folie a deux. On the subject of a case identified during confinement

**DOI:** 10.1192/j.eurpsy.2021.2115

**Published:** 2021-08-13

**Authors:** A. Alvarado Dafonte, L. Soldado Rodriguez, C. Coca Cruz

**Affiliations:** 1 Jaén, Complejo Hospitalario Jaén, Jaén, Spain; 2 Mental Health Unit, Complejo Hospitalario de Jaen, Jaen, Spain

**Keywords:** confinement, Folie a deux, Shared psychotic disorder

## Abstract

**Introduction:**

Shared psychotic disorder or Folie a deux is an unusual mental disorder characterized by the transfer of delusions between two or more people who have a close relationship. An individual (inductor or primary) who suffers from a psychotic disorder, influences one or more individuals (induced or secondary). Delusional disorders or schizophrenia are the most commonly diagnosed disorders in the inductor individual.

**Objectives:**

The objective of this study is to describe the clinical characteristics of an unusual entity such as shared psychotic disorder.

**Methods:**

Description of a clinical case of shared psychotic disorder of a family treated in the emergency room during confinement.

**Results:**

47-year-old woman, goes to the emergency room with her husband. No psychiatric history. Both the patient and her husband verbalize delusions of harm and surveillance from neighbors. They also report that two of their children hold this belief. The mother, unlike the rest of the cohabitants, presents disqualifying auditory hallucinations. Her husband decides to take her to the emergency room because he finds her distressed, “between two realities” and aggressive when she is confronted about hallucinations. We start treatment with oral paliperidone in the mother and a subsequent follow- up, and a total remission of symptoms in all cohabitants.

**Conclusions:**

As in other mental disorders, the correct diagnosis and subsequent referral are essential. The separation of the inductor individual from the induced one is useful for the correct management of this disorder. With timely intervention and a regular follow-up, the Folie a deux has a good prognosis.

**Disclosure:**

No significant relationships.

